# Are Lipid-Lowering and Antihypertensive Medications Used as Complements to Heart-Healthy Diets? A Scoping Review

**DOI:** 10.1016/j.advnut.2023.04.010

**Published:** 2023-04-29

**Authors:** Clémence Desjardins, Marie Cyrenne-Dussault, Olivier Barbier, Amélie Bélanger, Anne Gangloff, Line Guénette, Jacinthe Leclerc, Jean Lefebvre, Arsène Zongo, Jean-Philippe Drouin-Chartier

**Affiliations:** 1 Nutrition, Health and Society (NUTRISS) Research Center, Institute of Nutrition and Functional Foods (INAF), Laval University, Québec City, Canada; 2 Faculty of Pharmacy, Laval University, Québec City, Canada; 3 CHU de Québec-Université Laval Research Center, Québec City, Canada; 4 Faculty of Medicine, Laval University, Québec City, Canada; 5 Institut Universitaire de Cardiologie et de Pneumologie de Québec-Université Laval, Québec City, Canada

**Keywords:** cardiovascular disease prevention, lipid-lowering medication, antihypertensive medication, diet, scoping review

## Abstract

In cardiovascular disease (CVD) prevention, whether antihypertensive and lipid-lowering medications are used as complements to heart-healthy diets has not been thoroughly assessed. This scoping review aimed to *1*) analyze observational studies that assessed the relationship between diet and antihypertensive/lipid-lowering medication use and *2*) evaluate whether medication was used as a complement to heart-healthy dietary intakes. We searched MEDLINE, Embase, Web of Science, and CINAHL through 14 January, 2023, for studies that assessed either *1*) intraindividual changes in diet associated with lipid-lowering/antihypertensive medication initiation or use or *2*) interindividual differences in diet between users and nonusers of these medications. A total of 17 studies were included. Of those, 3 prospectively assessed the intraindividual changes in diet associated with medication initiation or use, but none documented potential changes in diet prior to medication initiation. The 14 other studies compared dietary intakes of medication users and nonusers, most of which also relied on an incomplete assessment of the temporal dynamics between diet and medication use as they employed cross-sectional (*n* = 12) or repeated cross-sectional (*n* = 2) designs. Data from 8 studies, including 4 of the 5 studies from Europe, suggested that medication was used as a complement to heart-healthy diets, whereas data from the 9 other studies, including the 4 conducted in the United States, provided no such evidence, indicating potential between-country differences in this relationship. Finally, no studies investigated how the dynamics between diet and medication use influenced the long-term CVD risk. This scoping review suggests that the current literature on the relationship between lipid-lowering/antihypertensive medication use and diet provides an incomplete perspective on how medication may influence diet in CVD prevention. Prospective studies assessing intraindividual changes in diet associated with medication initiation and use and how these dynamics influence the CVD risk are thus needed.

**Statement of Significance**

The current literature on the relationship between lipid-lowering and antihypertensive medication use and diet provides an incomplete perspective on how medication may influence diet in CVD prevention. Prospective studies assessing the changes in diet associated with medication use and how these dynamics influence the CVD risk are needed.

## Introduction

CVDs remain the leading cause of mortality and morbidity worldwide with a global economic burden expected to exceed $1 trillion by 2030 [1,2]. Dietary patterns low in red and processed meats, added sugar and sodium, and high in minimally processed plant foods have been demonstrated to have cardioprotective effects that are comparable to those of lipid-lowering or antihypertensive medications [[3], [4], [5], [6]]. Still, among individuals living with conditions that increase the CVD risk such as dyslipidemias or hypertension, the concurrent use of lipid-lowering and/or antihypertensive drugs while following a heart-healthy diet is indicated [1,7,8]. In such contexts, medication needs to be used as a complement to rather than a substitute for heart-healthy dietary modifications to maximize CVD risk reduction. In that regard, it has been suggested that the perception of high CVD risk associated with the action of initiating preventive medication is a potential facilitator to lifestyle modification, including diet [[9], [10], [11]]. However, qualitative studies reported that the perceived effectiveness of preventive medication, albeit being essential to pharmacotherapy adherence, may represent a barrier to diet improvements, leading users of these medications to maintain or even adopt unfavorable dietary habits [11,12]. This highlights the many factors with potentially conflicting influence on how preventive medication initiation or use may facilitate or impede dietary changes. As such, observational studies reported opposite observations with regard to diet changes in relation to medication initiation or use. For instance, a study conducted in Denmark in the early 2000s among individuals with hypertension or hypercholesterolemia showed that people who initiated medication targeting these CVD risk factors were more likely to improve their diet than those who remained untreated [13]. Conversely, data from the NHANES showed that, over the first decade of the 2000s in the United States, caloric and fat intakes consistently increased among individuals who used statins, whereas they remained stable among individuals who did not use statins [14]. Ultimately, if medication is used as a substitute for dietary management, CVD prevention is likely to remain suboptimal, whereas the risks of adverse events associated with medication use and those of medication overuse are likely to increase [15]. It is thus important to assess, from the existing literature, the relationship between preventive medication use and adherence to heart-healthy dietary habits to identify conditions that favor the complement approach. Such work is a crucial step to inform future research efforts and evidence-based policies to ensure that, in CVD prevention, when medication is indicated, it is systematically used as complement to rather than a substitute for heart-healthy dietary changes.

Therefore, we conducted a scoping review to identify and synthesize the observational literature on the relationship between the initiation or use of preventive medication, namely, lipid-lowering and antihypertensive drugs, and the diet. In this work, we aimed to answer the following questions:

1) What is known, from observational studies, on the relationship between lipid-lowering and antihypertensive medication initiation or use and dietary habits?
2) Based on previously published observational studies, are lipid-lowering and antihypertensive medications initiated or used as complements to heart-healthy diets?
3) What are the implications of the existing observational literature for future research?

## Methods

This work was conducted in an iterative fashion using the scoping study frameworks of Arksey and O’Malley [16] and Levac et al. [17]. It is reported according to the PRISMA extension for Scoping Reviews (PRISMA-ScR) guidelines [18]. The review protocol was registered in Open Science Framework [19].

We searched the literature for studies that provided information on either *1*) intraindividual changes in diet associated with lipid-lowering/antihypertensive medication initiation or use or *2*) interindividual differences in diet between users and nonusers of these medications. We searched MEDLINE (via PubMed), Embase, Web of Science, and CINAHL (via EbscoHost) through 14 January, 2023, using a combination of terms relevant to lipid-lowering/antihypertensive medication and diet. The specific search terms and search strategy for each database along with the number of records retrieved are presented in Supplemental Table 1. The search was restricted to studies published in English or French, but there was no restriction in terms of study design, population, or year of publication. EndNote X8 software was used to manage references.

Two bilingual authors (CD, MCD) independently screened titles and abstracts and, subsequently, full texts of potentially eligible studies identified with the search strategy. References cited in each included study were also screened to identify additional relevant studies. Disagreement and discordance between the 2 reviewers were resolved by discussion. When no agreement could be reached, the senior author (JPDC) was consulted. For each selected study, the following information was extracted: author’s name, year of publication, country where the study was conducted, study design, participants’ characteristics (sample size, sex, and age), duration of the study when applicable, type of medication initiated or used (lipid-lowering/antihypertensive), dietary assessment method, assessed dietary component(s) (overall diet quality and/or intakes of specific nutrients or foods), and changes in diet for studies that prospectively assessed intraindividual differences in diet associated with medication initiation or use or interindividual differences in diet for studies that compared medication users with nonusers. We also extracted information on whether the selected studies assessed the joint influence of diet and medication on plasma lipids, blood pressure, or CVD risk. Data extraction was performed in duplicate by 2 authors (CD, MCD) so that consensus could be reached on which data to extract. Disagreement and discordance were resolved by discussion. The senior author (JPDC) was consulted when no agreement could be obtained.

For data analysis, we first conducted a descriptive analysis of the pool of included studies. Studies were charted according to the year they were published, the country in which they were conducted, the type(s) of medication used or initiated by participants, the assessed dietary component(s) (that is, overall diet quality and intake of specific foods or nutrients), the comparative design (that is, intraindividual differences in diet associated with medication initiation or use or those in diet between medication users and nonusers), and the presence of an assessment of the joint influence of diet and medication use on CVD risk factors or CVD risk. Second, we provided a descriptive summary of each included study. Next, 2 authors (CD, JPDC) analyzed dietary data reported in each study to determine whether they reflected that medication was used as a complement to heart-healthy dietary changes or intakes over the study period [11,20]. We considered studies reporting that *1*) medication initiation or use was found to be associated with favorable dietary changes or *2*) dietary intakes of medication users were more favorable for cardiovascular health than those of nonusers to be reflective of the complement approach. This assessment was conducted in duplicates, and disagreements were resolved by discussion. Finally, we grouped the studies according to whether they were reflective of the complement approach or not. This step was used to identify potential between-study common characteristics associated with the complement approach.

## Results

**FIGURE 1:**
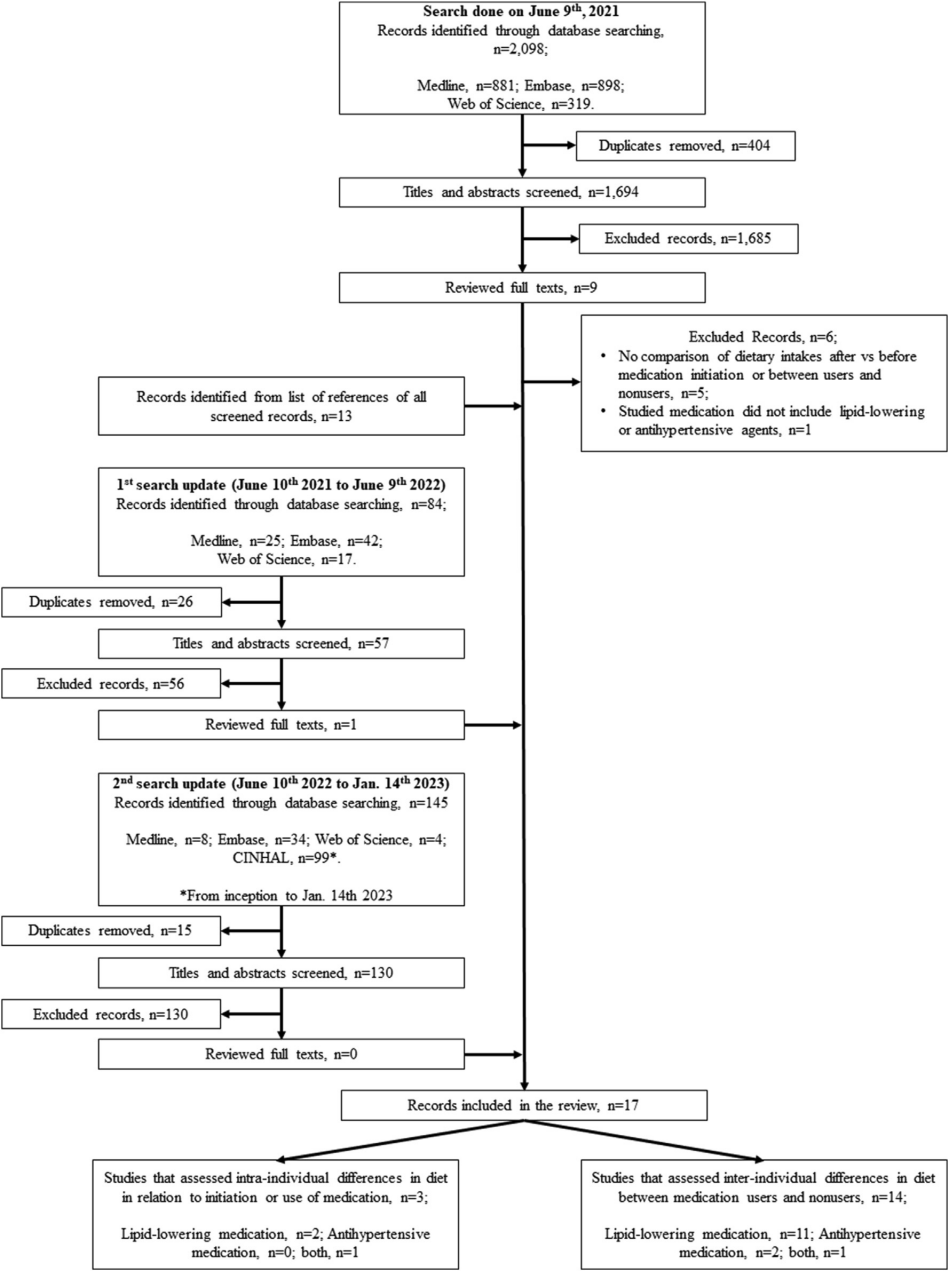
Flow chart of study selection.

We screened a total of 1881 publications and included 17 articles [9,13,14,[21], [22], [23], [24], [25], [26], [27], [28], [29], [30], [31], [32], [33], [34]] (Figure 1). The earliest publications were of the year 2007, and the most recent was published in 2021 (Table 1). Studies were conducted in the United States (*n* = 4), Australia (*n* = 3), Denmark (*n* = 2), Japan (*n* = 2), South Korea (*n* = 2), Guadeloupe (France overseas department) (*n* = 1), the Netherlands (*n* = 1), Sweden (*n* = 1), and the United Kingdom (*n* = 1). A total of 13 studies included individuals initiating or using lipid-lowering medication and 2 studies included individuals using antihypertensive medication, whereas the remaining 2 included individuals initiating or using lipid-lowering and/or antihypertensive drugs. In all included studies, unless otherwise specified in the text below, there was no mention on how long the treated individuals have been using medication prior to data collection or on whether nonusers had dyslipidemia and/or hypertension. Assessed dietary components included overall diet quality in 5 studies, intakes of foods in 8 studies, and nutrient intakes in 11 studies. In terms of comparative design, 3 studies prospectively assessed the intraindividual differences in diet associated with medication initiation or use, whereas the other 14 studies assessed interindividual differences in dietary habits between medication users and nonusers. Finally, only 2 studies assessed the joint influence of diet and medication on CVD risk factors [32,33], but none investigated how the dynamics between diet and medication influenced CVD incidence.

**TABLE 1:** Main characteristics of the 17 studies included in the scoping review

Study (reference)	Year	Country	Type of medication		Assessed dietary components			Comparative design	Assessment of the joint influence of diet and medication on CVD risk
			Lipid-lowering	Antihypertensive	Overall diet quality	Food intake	Nutrient intake		
Atallah et al. [23]	2007	Guadeloupe (France overseas department)		✓		✓		Interindividual differences between users and nonusers	No
Gadowski et al. [31]	2019	Australia	✓			✓		Interindividual differences between users and nonusers	No
Gadowski et al. [34]	2021	Australia	✓			✓		Interindividual differences between users and nonusers	No
Hempler et al. [13]	2012	Denmark	✓	✓	✓			Intraindividual differences associated with medication initiation	No
Hollestein et al. [27]	2015	Netherlands	✓		✓			Interindividual differences between users and nonusers	No
Johal et al. [29]	2017	Australia	✓				✓	Interindividual differences between users and nonusers	No
Kameyama et al. [33]	2020	Japan	✓			✓	✓	Interindividual differences between users and nonusers	Yes
Kanauchi et al. [26]	2015	Japan		✓	✓		✓	Interindividual differences between users and nonusers	No
Kinjo et al. [30]	2017	United States	✓	✓	✓		✓	Interindividual differences between users and nonusers	No
Lofgren et al. [24]	2010	United States	✓			✓	✓	Interindividual differences between users and nonusers	No
Lytsy et al. [9]	2012	Sweden	✓				✓	Interindividual differences between users and nonusers	No
Mann et al. [21]	2007	United States	✓			✓	✓	Intraindividual differences associated with medication initiation	No
McAleer et al. [22]	2016	United Kingdom	✓			✓	✓	Intraindividual differences associated with medication use	No
Oh et al. [28]	2016	South Korea	✓				✓	Interindividual differences between users and nonusers	No
Sugiyama et al. [14]	2014	United States	✓				✓	Interindividual differences between users and nonusers	No
Thomsen et al. [25]	2013	Denmark	✓		✓			Interindividual differences between users and nonusers	No
Yang et al. [32]	2019	South Korea	✓			✓	✓	Interindividual differences between users and nonusers	Yes

Table 2 presents data from the 3 studies that prospectively assessed intraindividual changes in diet associated with medication initiation or use. Of these, 2 assessed whether diet changed over the first months of statin initiation [21,22]. Mann et al. [21] evaluated diet at the time of statin prescription and after 6 mo among 62 adults from the United States, all of whom initiated such therapy. No evidence of changes in diet was reported. Similarly, McAleer et al. [22] assessed diet 1 mo and 4 mo after lipid-lowering medication initiation among 109 adults from the United Kingdom. No evidence of differences in diet was also observed. In the third study, Hempler et al. [13] evaluated changes in diet over a 5-y period in relation with lipid-lowering or antihypertensive medication initiation among 871 adults from the Inter99 cohort in Denmark. No participants were using medication at baseline, but 292 (34%) and 408 (47%) initiated lipid-lowering or antihypertensive medication, respectively, at some point during the follow-up. Diet was assessed at baseline and at the 5-y follow-up, but not at the moment of medication prescription. Among participants who initiated medication, the proportion whose diet quality improved was greater than the proportion whose diet quality remained unchanged or deteriorated.

**TABLE 2:** Studies that assessed intraindividual differences in diet in relation to initiation or use of lipid-lowering and/or antihypertensive medication

Study (reference)	Participants (*n*)	Age (y)	Study design	Dietary assessment method	Initiated medication	Assessed dietary components and reported results
Mann et al. [21]	71 (male: *n* = 64; 90%; female: *n* = 7; 10%); 62 completed the dietary recall at 6 mo	Mean ± SD: 61.0 ± 12.6	Prospective: 6 mo of follow-up in 2005	- Interviewer administered 24-h dietary recall (standardized USDA automated multiple-pass method) at baseline and follow-up - Changes in dietary intakes 6 mo after medication initiation vs. before medication (study baseline) were assessed.	Lipid-lowering	Total energy, Δ = −129 kcal/d (*P* = 0.20)
						Total fat, Δ = −2 g/d (*P* = 0.38)
						SFA, Δ = −2 g/d (*P* = 0.19)
						MUFA, Δ = 0 g/d (*P* = 0.59)
						PUFA, Δ = +1 g/d (*P* = 0.16)
						Fruits and vegetables, Δ = +1 serving/d (*P* = 0.35)
						Dietary fiber, Δ = +1 g/d (*P* = 0.08)
McAleer et al. [22]	109 (male: *n* = 50; 45.9%; female: *n* = 59; 54.1%)	Mean ± SD: 64.0 ± 10.3	Prospective: 4 mo of follow-up in 2005	- 4-d food diary complemented with a 19-item interviewer-administered FFQ - Changes in dietary intakes 4 mo after medication initiation vs. 1 mo after medication initiation (study baseline) were assessed.	Lipid-lowering	Total fat (% of total energy), Δ = −0.54% (95% CI: −1.72, 0.64)
						SFA (% of total energy), Δ = −0.67% (95% CI: −1.44, 0.10)
						MUFA (% of total energy), Δ = +0.30% (95% CI: −0.13, 0.74)
						PUFA (% of total energy), Δ = +0.37% (95% CI: −0.01, 0.75)
						Dietary fiber (g/d), Δ = +0.23 (95% CI: −0.61, 1.07)
						Fruits and vegetables (serving/d), Δ = +0.00 (95% CI: −0.21, 0.20)
Hempler et al. [13]	871 (male: *n* = 507; 58.2%; female: *n* = 364; 41.8%)	Range (min to max): 30–60	Prospective: 5 y of follow-up over 1999–2006	- Self-administrated 52-item validated FFQ - Diet quality was assessed using a score based on intakes of fish, vegetables, fruits, and fat - The prevalence of individuals with a higher diet quality score at 5 y of follow-up was compared to those with unchanged/unhealthier diet among individuals who initiated medication during the follow-up - The odds of having a higher diet quality score at 5 y of follow-up vs. baseline associated with the initiation of medication were also assessed.	Lipid-lowering	Individuals using lipid-lowering medication with a healthier diet vs. individuals using lipid-lowering medication with an unhealthier/unchanged diet at 5-y follow-up = 31.5% vs. 20.8% (*P* = 0.04), respectively
						OR (95% CI) of having a healthier diet at 5 y associated with the initiation of lipid-lowering medication: 2.08 (1.03, 4.21)
					Antihypertensive	Individuals using antihypertensive medication with a healthier diet vs. individuals using antihypertensive medication with an unhealthier/unchanged diet at 5-y follow-up = 55.3% vs. 42.3% (*P* = 0.009), respectively
						OR (95% CI) of having a healthier diet at 5 y associated with the initiation of antihypertensive medication: 1.58 (0.96, 2.59)

Abbreviations: min, minimum; max, maximum.

Data from the 14 studies that assessed interindividual differences in dietary habits between lipid-lowering or antihypertensive medication users and nonusers are presented in Table 3. Of these, 10 compared the dietary intakes of lipid-lowering or antihypertensive medication users and nonusers using a cross-sectional design. Specifically, Lofgren et al. [24] compared the diet of 115 adults from the United States who were using statins (*n* = 37) or not (*n* = 78). No evidence of difference in diet between statin users and nonusers was reported, with the exception that users consumed less vegetables, calcium, and potassium than nonusers. Thomsen et al. [25] compared the overall diet quality of 13,787 Danish individuals who were either using statins (*n* = 1641) or not (*n* = 12,146). Statin users were more likely to have a “healthy diet” and less likely to have an “unhealthy diet” than nonusers, although no definition of “healthy/unhealthy” diets was provided in the original article. Kanauchi et al. [26] compared the overall diet quality as well as energy and sodium intakes of 433 Japanese men who had either treated hypertension (antihypertensive users, *n* = 37), untreated hypertension (antihypertensive nonusers with hypertension, *n* = 97), or no hypertension (antihypertensive nonusers without hypertension, *n* = 299). No evidence of difference in diet quality and in intakes of energy and sodium was found between the 3 groups. Hollestein et al. [27] compared diet quality of 705 individuals using statins to that of 7046 individuals not using statins from the Rotterdam cohort study. The diet quality was assessed using the Dutch Healthy Diet score, for which higher scores reflect greater adherence to the Netherlands’ dietary guidelines. Statin users were more likely to be within the highest tertile of the score. Johal et al. [29] compared saturated fat intake of 1108 statin users to that of 3506 nonusers from Australia. Statin users were less likely to have saturated fat intake within the highest quartile than nonusers. Kinjo et al. [30] compared the diet quality between individuals taking antihypertensive medication (*n* = 8099) and individuals with untreated hypertension (*n* = 3752), as well as between individuals using lipid-lowering medication (*n* = 4645) and individuals with untreated dyslipidemia (*n* = 4550), from the United States by pooling the 1999–2010 NHANES data. The DASH score was used to assess diet quality (higher scores reflect higher diet quality). Individuals using antihypertensive medication had higher intakes of total fat, cholesterol, protein, calcium, magnesium, and sodium, and were not more likely to have a DASH score >4.5/9.0 than individuals with untreated hypertension. Similarly, individuals using lipid-lowering medication had higher intakes of magnesium, calcium, and sodium, and were not more likely than individuals with untreated dyslipidemia to have a DASH score >4.5/9.0. Gadowski et al. [34] compared dietary intakes of community-dwelling older adults with dyslipidemia using lipid-lowering medication (*n* = 72) with those not using lipid-lowering medication (*n* = 38) from Australia. There was no evidence of difference in adherence to Australian dietary guidelines between the 2 groups. The same group of authors used data from the Australian Diabetes, Obesity, and Lifestyle Study to compare adherence to Australian dietary recommendations of 446 individuals using lipid-lowering medication and 4813 individuals who were not using lipid-lowering medication, both at the study baseline and at the 5-y follow-up [31]. Individuals using lipid-lowering medication were treated from baseline until the end of the 5-y follow-up, and those who were not using medication at baseline remained untreated over the same period. The only difference in adherence to Australian dietary guidelines between the 2 groups was that medication users were less likely to meet the recommendation for protein intake than nonusers, both at baseline and at the 5-y follow-up. Yang et al. [32] used the 2015–2017 KNHANES data to compare the diet of individuals with dyslipidemia using lipid-lowering medication (*n* = 1734) with that of individuals with dyslipidemia not using lipid-lowering medication (*n* = 856) in a cross-sectional fashion. There was no evidence of differences in dietary intakes between the 2 groups. Additionally, in this study, the joint influence of dietary intakes and lipid-lowering medication use on total plasma cholesterol levels was investigated. With the exception of an inverse association between sugar intake and cholesterol levels among nonusers, no evidence of a differential association between diet and cholesterol levels, per medication use status, was observed. Still, no interaction analyses were conducted to determine whether dietary intakes influenced the relationship between medication use and plasma cholesterol concentrations. Finally, Kameyama et al. [33] compared dietary intakes of Japanese individuals with dyslipidemia who had been using lipid-lowering medication for ≥3 mo (*n* = 45) with those of individuals with dyslipidemia not using medication (*n* = 59). Individuals using lipid-lowering medication had lower intakes of meat, poultry, and processed meat products and higher intakes of fish, fruits, omega-3 PUFAs, EPA, and DHA than nonusers. The joint influence of diet and medication on plasma LDL-C levels was also assessed in this study. On one hand, lipid-lowering medication use was associated with lower LDL-C levels, as expected. On the other hand, intakes of seafoods, sweets, animal fats, and hydrogenated margarine were positively associated with LDL-C. However, no interaction analyses were conducted to determine whether dietary intakes influenced the relationship between medication use and LDL-C concentrations.

**TABLE 3:** Studies that assessed interindividual differences in diet between users and nonusers of lipid-lowering and/or antihypertensive medication

Study (reference)	Participants (*n*)	Age (y)	Study design	Dietary assessment method	Comparison groups	Assessed dietary components	Users	Nonusers	*P* value
Lofgren et al. [24]	115 (male: *n* = 33; 28.7%; female: *n* = 82; 71.3%)	Mean ± SD: 75.2 ± 6.8	Cross-sectional	- Three 24-h dietary recalls	- Users: individuals with dyslipidemia using lipid-lowering medication (statins), *n* = 37 (32.2%) - Nonusers: individuals not using lipid-lowering medication, *n* = 78 (67.8%)	Total energy (kcal/d ± SD)	1458 ± 460	1570 ± 455	NS
						Fruits (servings/d ± SD)	2.3 ± 1.3	2.7 ± 1.3	NS
						Vegetables (servings/d ± SD)	2.5 ± 1.6	3.1 ± 1.4	<0.05
						Carbohydrates (% of energy ± SD)	54.5 ± 8.3	55.7 ± 7.8	NS
						Proteins (% of energy ± SD)	17.3 ± 3.9	16.9 ± 3.7	NS
						Total fat (% of energy ± SD)	29.7 ± 8.3	28.9 ± 6.6	NS
						SFA (% of energy ± SD)	9.5 ± 3.6	9.4 ± 2.9	NS
						MUFA (% of energy ± SD)	11.3 ± 3.6	10.9 ± 2.7	NS
						PUFA (% of energy ± SD)	6.3 ± 2.1	6.1 ± 2.1	NS
						TFA (g/d ± SD)	3.2 ± 1.9	3.6 ± 2.0	NS
						Cholesterol (mg/d ± SD)	203 ± 143	226 ± 143	NS
						n-3 PUFA (g/d ± SD)	1.3 ± 0.8	1.3 ± 0.6	NS
						Dietary fiber (g/d ± SD)	15.8 ± 7.3	17.7 ± 6.5	NS
						Sodium (mg/d ± SD)	2358 ± 954	2468 ± 865	NS
						Calcium (mg/d ± SD)	613.3 ± 260.2	753.7 ± 356.8	<0.05
						Potassium (mg/d ± SD)	2408 ± 899	2779 ± 807	<0.05
Thomsen et al. [25]	13,787	Range (min to max): 25–79	Cross-sectional	- 30-item FFQ	- Users: individuals with dyslipidemia using lipid-lowering medication (statins), *n* = 1641 (11.7%) - Nonusers: individuals not using lipid-lowering medication, *n* = 12,146 (86.8%).	PR (95% CI) of having a “healthy diet” (no definition provided)	1.43 (1.30–1.56)	1.00 (reference)	<0.05
						PR (95% CI) of having a “reasonably healthy diet” (no definition provided)	0.91 (0.86–0.95)	1.00 (reference)	<0.05
						PR (95% CI) of having a “unhealthy diet” (no definition provided)	0.70 (0.59–0.84)	1.00 (reference)	<0.05
Kanauchi et al. [26]	433 (male: *n* = 433; 100%)	Mean ± SD: 45.3 ± 7.0	Cross-sectional	- Self-administered 58-item FFQ	- Users: individuals with hypertension using antihypertensive medication, *n* = 37 (8.5%) - Nonusers: individuals with hypertension not using antihypertensive medication, *n* = 97 (22.4%)	Energy (kcal/d ± SD)	1871 ± 489	1978 ± 560	NS
						Salt (g/1000 kcal ± SD)	5.63 ± 1.35	5.91 ± 1.26	NS
						HDI score ± SD	4.27 ± 1.02	4.12 ± 1.24	NS
						AI-84 score ± SD	27.7 ± 8.8	24.6 ± 10.1	NS
						DASH score ± SD	2.70 ± 1.13	2.38 ± 1.21	NS
						MED score ± SD	5.51 ± 1.35	5.41 ± 1.78	NS
					- Users: individuals with hypertension using antihypertensive medication, *n* = 37 (8.5%) - Nonusers: individuals without hypertension, *n* = 299 (69.1%)	Energy (kcal/d ± SD)	1871 ± 489	1923 ± 540	NS
						Salt (g/1000 kcal ± SD)	5.63 ± 1.35	5.86 ± 1.33	NS
						HDI score ± SD	4.27 ± 1.02	4.59 ± 1.16	NS
						AI-84 score ± SD	27.7 ± 8.8	27.3 ± 9.8	NS
						DASH score ± SD	2.70 ± 1.13	2.45 ± 1.31	NS
						MED score ± SD	5.51 ± 1.35	5.24 ± 1.45	NS
Hollestein et al. [27]	7751	≥20 [44]	Cross-sectional	- Validated 170-item semiquantitative FFQ	- Users: individuals with dyslipidemia using lipid-lowering medication (statins), *n* = 705 (9.1%) - Nonusers: individuals not using lipid-lowering medication, *n* = 7046 (90.9%)	OR (95% CI) of healthy diet (defined as the first tertile in DHD score)	1.32 (1.05–1.67)	1.00 (reference)	NS
Johal et al. [29]	4614 (male: *n* = 2062; 44.7%; female: *n* = 2552; 55.3%)	Mean ± SD: users: 67.8 ± 9.7; nonusers: 58.6 ± 10.9	Cross-sectional	- Self-administered FFQ	- Users: individuals with dyslipidemia using lipid-lowering medication (statins), *n* = 1108 (24%) - Nonusers: individuals not using lipid-lowering medication, *n* = 3506 (76%)	OR (95% CI) of having saturated fat intake within the highest quartile	0.71 (0.54–0.94)	1.00 (reference)	NA
Kinjo et al. [30]	14,856 (male: *n* = 7324; 49.3%; female: *n* = 7532; 50.7%)	Mean: 62.2 Range (min to max): 20–85	Cross-sectional (pooling of 1999–2010 NHANES cycles)	- Interviewer-led 24-h dietary recall - 1999–2001: computer-assisted automated data collection system with a multiple-pass format - 2002–2010: USDA dietary data collection instrument, the automated multiple-pass method	- Users: individuals with hypertension using antihypertensive medication, *n* = 8099 (38.5%) - Nonusers: individuals with hypertension not using antihypertensive medication, *n* = 3752 (17.8%)	OR (95% CI) of DASH score of >4.5/9	1.0 (0.9–1.1)	1.00 (reference)	0.70
						OR (95% CI) of having a total fat intake of <27% of energy	0.9 (0.8–1.0)	1.00 (reference)	0.02
						OR (95% CI) of having a SFA intake of <6% of energy	0.9 (0.9–1.0)	1.00 (reference)	0.20
						OR (95% CI) of having a total protein intake of >18% of energy	1.1 (1.0–1.3)	1.00 (reference)	0.01
						OR (95% CI) of having a fiber intake <14.8 g/1000 kcal	1.0 (0.9–1.1)	1.00 (reference)	0.90
						OR (95% CI) of having a cholesterol intake of <71.4 mg/1000 kcal	0.9 (0.8–1.0)	1.00 (reference)	0.01
						OR (95% CI) of having a calcium intake of >590 mg/1000 kcal	1.2 (1.0–1.3)	1.00 (reference)	0.03
						OR (95% CI) of having a magnesium intake of >238 mg/1000 kcal	1.3 (1.1–1.6)	1.00 (reference)	0.009
						OR (95% CI) of having a potassium intake of >2238 mg/1000 kcal	1.1 (1.0–1.2)	1.00 (reference)	0.20
						OR (95% CI) of having a sodium intake of <1143 mg/1000 kcal	0.8 (0.7–0.9)	1.00 (reference)	<0.001
					- Users: individuals with dyslipidemia using lipid-lowering medication, *n* = 4645 (22.1%) - Nonusers: individuals with dyslipidemia not using lipid-lowering medication, *n* = 4550 (21.6%)	OR (95% CI) of DASH score of >4.5/9	1.0 (0.8–1.1)	1.00 (reference)	0.90
						OR (95% CI) of having a total fat intake of <27% of energy	1.0 (0.9–1.1)	1.00 (reference)	0.90
						OR (95% CI) of having a SFA intake of <6% of energy	1.0 (0.8–1.2)	1.00 (reference)	0.80
						OR (95% CI) of having a total protein intake of >18% of energy	0.9 (0.8–1.0)	1.00 (reference)	0.05
						OR (95% CI) of having a fiber intake of <14.8 g/1000 kcal	1.0 (0.9–1.1)	1.00 (reference)	0.50
						OR (95% CI) of having a cholesterol intake of <71.4 mg/1000 kcal	1.0 (0.9–1.1)	1.00 (reference)	0.90
						OR (95% CI) of having a calcium intake of >590 mg/1000 kcal	1.2 (1.1–1.3)	1.00 (reference)	0.03
						OR (95% CI) of having a magnesium intake of >238 mg/1000 kcal	1.0 (0.9–1.1)	1.00 (reference)	0.90
						OR (95% CI) of having a potassium intake of >2238 mg/1000 kcal	1.0 (0.9–1.1)	1.00 (reference)	0.70
						OR (95% CI) of having a sodium intake of <1143 mg/1000 kcal	0.9 (0.8–1.0)	1.00 (reference)	0.03
Gadowski et al. [31]	5895 (male: *n* = 2692; 45.7%; female: *n* = 3203; *n* = 54.3%)	≥25	Cross-sectional with 2 measurement periods (1999 and 2005)	- Self-administered 121-item FFQ, recalling food intake over the previous 12 mo - Dietary intakes at baseline and after 5 y were compared to the Australian Dietary Guidelines	- Users: individuals with dyslipidemia using lipid-lowering medication, *n* = 446 (7.6%) - Nonusers: individuals not using lipid-lowering medication, *n* = 4813 (81.6%).	Individuals meeting guidelines for all food groups at baseline (%)	0.5	0.2	NS
						Individuals meeting guidelines for vegetables at baseline (%)	2.9	2.4	NS
						Individuals meeting guidelines for fruits at baseline (%)	28.3	26.0	NS
						Individuals meeting guidelines for cereals at baseline (%)	24.4	18.7	NS
						Individuals meeting guidelines for protein at baseline (%)	81.8	87.7	0.001
						Individuals meeting guidelines for dairy at baseline (%)	20.2	21.0	NS
						Individuals meeting guidelines for all food groups at year 5 (%)	0.2	0.1	NS
						Individuals meeting guidelines for vegetables at year 5 (%)	1.4	2.0	NS
						Individuals meeting guidelines for fruit at year 5 (%)	26.3	28.5	NS
						Individuals meeting guidelines for cereal at year 5 (%)	17.0	14.1	NS
						Individuals meeting guidelines for protein at year 5 (%)	81.6	87.5	0.001
						Individuals meeting guidelines for dairy at year 5 (%)	19.3	20.0	NS
Yang et al. [32]	2590 (male: *n* = 940; 36.3%; female: *n* = 1650; 63.7%)	Mean ± SD: users: 61.8 ± 0.4; nonusers: 55.0 ± 0.5	Cross-sectional (pooling of 2015–2017 KNHANES cycles)	- 24-h recall administered during face-to-face interviews.	- Users: individuals with dyslipidemia using lipid-lowering medication, *n* = 1734 (66.9%); - Nonusers: individuals with dyslipidemia not using lipid-lowering medication, *n* = 856 (33.1%).	Energy (kcal/d, mean ± SE)	1960 ± 39	1790 ± 26	0.46
						Cereals (g/d ± SE)	290 ± 7	275 ± 4	0.61
						Sugar (g/d ± SE)	11.2 ± 0.9	9.0 ± 0.4	0.35
						Vegetables (g/d ± SE)	346 ± 9	335 ± 7	0.91
						Fruits (g/d ± SE)	210 ± 11	225 ± 9	0.19
						Meat (g/d ± SE)	97.5 ± 6.8	74.7 ± 4.1	0.69
						Eggs (g/d ± SE)	29.5 ± 2.3	23.4 ± 1.2	0.39
						Fish (g/d ± SE)	106 ± 7	103 ± 6	0.73
						Milk (g/d ± SE)	75.2 ± 5.4	75.1 ± 4.0	0.31
						Proteins (g/d ± SE)	71.0 ± 1.8	63.1 ± 1.2	0.74
						Total fat (g/d ± SE)	43.4 ± 1.4	35.6 ± 0.9	0.80
						SFA (g/d ± SE)	12.9 ± 0.5	10.2 ± 0.3	0.41
						MUFA (g/d ± SE)	13.9 ± 0.5	11.0 ± 0.3	0.62
						PUFA (g/d ± SE)	10.9 ± 0.3	9.6 ± 0.2	0.36
						n-3 PUFA (g/d ± SE)	1.8 ± 0.1	1.8 ± 0.1	0.18
						n-6 PUFA (g/d ± SE)	9.1 ± 0.3	7.8 ± 0.2	0.58
						Cholesterol (mg/d ± SE)	250 ± 12	202 ± 8	0.67
						Carbohydrates (g/d ± SE)	513 ± 13	295 ± 4	0.46
						Dietary fiber (g/d ± SE)	26.3 ± 0.6	25.9 ± 0.4	0.56
Kameyama et al. [33]	104 (male: *n* = 51; 49%; female: *n* = 53; 51%)	Mean ± SD: 53.0 ± 8.0	Cross-sectional	- Self-completed 3-d weighted dietary record	- Users: individuals with dyslipidemia using lipid-lowering medication, *n* = 45 (43.3%) - Nonusers: individuals with dyslipidemia not using lipid-lowering medication, *n* = 59 (56.7%)	Energy (kcal/d)	1927	1869	0.71
						Refined cereals (g/1000 kcal)	173.4	183.1	0.11
						Unrefined cereals (g/1000 kcal)	0.0	8.6	0.07
						Meat, poultry, and processed meat products (g/1000 kcal)	44.0	51.3	0.01
						Eggs (g/1000 kcal)	15.5	17.6	0.63
						Milk and dairy products (g/1000 kcal)	44.3	34.9	0.30
						Fish (g/1000 kcal)	30.2	16.1	0.004
						Vegetables (g/1000 kcal)	131.6	151.4	0.90
						Fruits (g/1000 kcal)	37.8	7.1	0.001
						Animal fats, SFA-rich vegetable oils, and margarine (g/1000 kcal)	0.6	0.6	0.85
						Sugar-sweetened beverages (g/1000 kcal)	0.5	0.0	0.37
						Total fat (% of energy)	29.8	29.9	0.35
						SFA (% of energy)	8.5	8.7	0.74
						MUFA (g/d)	23.3	24.4	0.55
						n-3 PUFA (g/d)	2.7	2.2	0.006
						EPA+DHA (g/d)	0.8	0.4	0.002
						n-6 PUFA (g/d)	10.7	11.0	0.89
						Cholesterol (mg/d)	333	346	0.66
						Carbohydrates (% of energy)	54.6	55.6	0.86
						Dietary fiber (g/1000 kcal)	7.9	7.5	0.25
						Proteins (% of energy)	15.3	14.7	0.22
						Salt equivalent (g/d)	10.2	9.6	0.96
Gadowski et al. [34]	110 (male: *n* = 57; 51.8%; female: *n* = 53; *n* = 48.2%)	Mean ± SD: 76.8 ± 4.5 Range (min to max): 71–94	Cross-sectional	- Self-administered validated 6-item FFQ, recalling food intake over the previous 12 mo - Dietary intakes were compared to the NHMRC 2013 Australian Dietary Guidelines.	- Users: individuals with dyslipidemia using lipid-lowering medication, *n* = 72 (65.5%) - Nonusers: individuals not using lipid-lowering medication, *n* = 38 (34.5%).	Individuals meeting guidelines for vegetables (%)	2.8	2.6	NS
						Individuals meeting guidelines for fruits (%)	33.3	39.5	NS
						Individuals meeting guidelines for cereals (%)	16.7	18.4	NS
						Individuals meeting guidelines for protein (%)	29.2	31.6	NS
						Individuals meeting guidelines for dairy (%)	1.4	2.6	NS
Atallah et al. [23]	509	≥35	Cross-sectional	- Computer-assisted telephone interview - Self-reported perceived changes in dietary intakes over the year preceding the assessment	- Users: individuals with hypertension using antihypertensive medication, *n* = 163 (32%) - Nonusers: individuals without hypertension, *n* = 346 (68%)	Individuals that reported having decreased their cheese intake (%)	11	8	NA
						Individuals that reported having decreased their processed meat intake (%)	23	16	NA
						Individuals that reported having increased their fruit and vegetable intake (%)	29	46	NA
Lytsy et al. [9]	1458 (male: *n* = 756; 51.9%; female: *n* = 702; 71.3%)	Range (min to max): 40–80	Cross-sectional	Intentions regarding dietary habits were assessed with 2 questions: - “Do you try to avoid fatty foods?” - “Do you try to eat foods with a high fiber content, such as wholegrain bread, muesli or root vegetables?”	- Users: individuals with dyslipidemia using lipid-lowering medication (statins), *n* = 829 (56.9%) - Nonusers: individuals not using lipid-lowering medication, *n* = 629 (43.1%)	OR (95% CI) of trying to avoid eating foods with high fat content (%)	2.33 (1.59, 3.42)	1.00 (reference)	<0.01
						OR (95% CI) of trying to eat foods with high fiber content	1.58 (1.15, 2.17)	1.00 (reference)	<0.01
Sugiyama et al. [14]	Cycle specific (min, *n* = 4594; max, *n* = 6149)	≥20	Repeated cross-sectional; 11 y (1999–2010)	- Interviewer-led 24-h dietary recall - 1999–2001: computer-assisted automated data collection system with a multiple-pass format - 2002–2010: USDA dietary data collection instrument, the automated multiple-pass method	- Users: individuals with dyslipidemia using lipid-lowering medication (statins) - Nonusers: individuals not using lipid-lowering medication	11-y change in total energy intake (%)	+9.6 (95% CI: 1.8, 18.1)	−1.9 (95% CI: −4.6, 0.9)	0.001
						11-y change in total fat intake (%)	+14.4 (95% CI: 3.8, 26.1)	−2.3 (95% CI: −5.6, 1.1)	<0.001
Oh et al. [28]	2635 (male: *n* = 1133; 43%; female: *n* = 1502; 57%)	Mean ± SD: 58.5 ± 0.31	Repeated cross-sectional; 3 y (2010–2013)	24-h dietary recall administered during face-to-face interviews	- Users: individuals with dyslipidemia using lipid-lowering medication, *n* = 1562 (56.6%) - Nonusers: individuals with dyslipidemia not using lipid-lowering medication, *n* = 1073 (43.4%)	3-y change in energy intake (%)	−7.02 (*P* = 0.10)	+6.45 (*P* = 0.97)	NA

Abbreviations: AI-84, adherence index with a maximum possible score of 84; DHD, Dutch diet index; HDI, healthy diet index; KNHANES, Korean National Health and Nutrition Examination Survey; max, maximum; MED, Mediterranean diet; min, minimum; n-3, omega-3; n-6, omega-6; NA, not applicable; NHMRC, National Health and Medical Research Council; PR, prevalence ratio; TFA, trans fatty acids.

The next 2 studies included in this review did not objectively assess diet intakes per se (Table 3). Indeed, Atallah et al. [23] assessed the self-perceived changes in diet over the year preceding data collection among 509 adults from Guadeloupe with treated hypertension (*n* = 163) or without hypertension (*n* = 346). Individuals using antihypertensive medication were more likely to report decreased cheese and processed meat intakes, but less likely to report increased fruit and vegetable intakes than those without hypertension. Lytsy et al. [9] assessed the intentions of avoiding foods high in fat and consuming foods high in dietary fiber among 1458 Swedish adults who were using statins (*n* = 829) or not (*n* = 629). Users more likely avoided foods high in fat and ate foods high in fiber than nonusers.

Finally, we identified 2 studies that compared trends in dietary intakes between statin users and nonusers using repeated cross-sectional data from national health surveys (Table 3). Sugiyama et al. [14] compared trends in energy and total fat intakes between statin users and nonusers from the United States using repeated cross-sectional data from the 1999–2010 NHANES. Caloric and fat intakes both increased over time among individuals using statins, but not among nonusers. Oh et al. [28] conducted a similar analysis among 1562 statin users and 1073 nonusers with dyslipidemia using the 2010–2013 Korea NHANES (KNHANES). A statistical trend suggested that energy intake decreased over the 3-y period among statin users, but no evidence of change was observed among nonusers. Differences in the 2 trends were not compared.

Overall, observations reported in 8 of the 17 reviewed studies were reflective of the complement approach [9,13,23,25,27,29,31,33], that is, medication initiation was associated with favorable dietary changes, or diet habits of medication users were more favorable for cardiovascular health than those of nonusers. Specifically, 4 out of the 5 studies taking place in Europe [9,13,25,27], including the 2 from Denmark [13,25], were suggestive of the complement approach, whereas data from the 4 studies in the United States provided no evidence of such approach [14,21,24,30].

## Discussion

In this scoping review on the relationship between the initiation or use of lipid-lowering/antihypertensive medication and diet, we aimed *1*) to determine, from the existing observational literature, what is known on this relationship per se and *2*) to evaluate whether lipid-lowering/antihypertensive medications were used as complements to heart-healthy diets in identified studies. Of the 17 studies we reviewed, 3 prospectively assessed intraindividual changes in diet associated with medication initiation or use. The other 14 compared the diet of medication users and nonusers using cross-sectional or repeated cross-sectional designs. Additionally, no studies documented potential changes in diet prior to medication initiation or assessed how the dynamics between diet and medication use influence CVD incidence. With regard to how medication appeared to have been used relative to diet, data from 8 studies, including 4 of the 5 studies from Europe, reflected the complement approach, whereas the 9 other studies, including the 4 conducted in the United States, provided no evidence of such approach. Overall, this work sheds light on the limited scope of the current literature on the relationship between lipid-lowering/antihypertensive medication use and diet while identifying the potential between-country differences underlying this relationship. As such, prospective studies characterizing long-term dynamics between diet quality, medication initiation and use, and CVD incidence are needed to understand the long-term implications of suboptimal adequacy between dietary and pharmaceutical management. Furthermore, cross-country comparisons are needed to identify systemic facilitators and barriers to the complement approach.

CVD prevention clinical guidelines worldwide recommend initiating lifestyle management, of which diet is a key component, and medication sequentially [1,7,8]. Indeed, medication is indicated when lifestyle modification is found to induce insufficient improvements in blood lipids or blood pressure or if dyslipidemia and/or hypertension are too severe upon diagnosis [1,7,8]. Therefore, to adequately capture the relationship between lipid-lowering/antihypertensive medication use and diet, it appears crucial to take into account the underlying temporal dynamics and assess diet in the period preceding medication initiation, at the moment of prescription, and during the period following medication initiation. The importance of such temporal consideration relies on the strong relationship between lifetime cumulative exposure to risk factors and CVD incidence [35]. Still, of the 17 studies we identified and reviewed, none had a design providing such information. Additionally, in most studies, there was no mention of whether individuals not using medication had dyslipidemia and/or hypertension, whether these individuals were aware that they might have dyslipidemia and/or hypertension, and whether the study participants received dietary/lifestyle counseling prior to data collection. Thus, on an individual basis, the studies we reviewed in this work provided very limited information on the potential facilitators of the complement approach. By the same extent, little can be inferred on the conditions that may lead to a situation where medication is used as a substitute for dietary improvements. As such, our work highlights that the current literature provides an incomplete perspective on how medication initiation or use may influence diet and how this relationship influences CVD risk in the long term. This is an important issue to assess considering that over the past 2 decades in the United States and worldwide, the prevalence of both lipid-lowering and antihypertensive medication use has drastically increased [36,37] while the population’s diet quality has remained mostly stable and suboptimal and CVD has remained a leading cause of mortality worldwide [38]. As such, prospective studies assessing intraindividual changes in diet associated with medication initiation or use and how these dynamics impact CVD incidence are crucially needed. Studies with such design have been published in the recent years and provided highly relevant data with regard to clinical practice and public health on the interrelationships between medication initiation and tobacco smoking, physical activity practice, or alcohol drinking. For instance, a large Finnish cohort study reported that lipid-lowering and antihypertensive medication initiation induced favorable declines in tobacco smoking and alcohol consumption, but also unfavorable changes in physical activity practice [11]. Similarly, a United States study reported that the weight gain associated with smoking cessation does not mitigate the long-term health benefits of quitting smoking even though it is linked to increased risks of dyslipidemia, hypertension, or diabetes in the short term [39]. Although not directly related to medication initiation, the latter study shows the clinical and public health relevance of prospectively assessing the benefits associated with co-occurring changes in cardiopreventive strategies.

Although we consider that, on an individual perspective, the studies we reviewed provided little information on the conditions that may favor the complement approach, collectively, these studies shed light on apparent between-country differences in the diet–medication relationship. These observations are hypothesis-generating and should inform future research. Indeed, 4 out of the 5 studies taking place in Europe [9,13,25,27], including the 2 from Denmark [13,25], were suggestive of the complement approach, whereas data from the 4 United States studies we reviewed provided no evidence of such approach [14,21,24,30]. In the United States, costs of healthcare represent a barrier to lifestyle medicine, especially considering that ∼1 in 10 adults do not have medical insurance, whereas in Denmark, the universal healthcare system may facilitate access to lifestyle medicine [40,41]. Moreover, a recent comparative analysis of European and American guidelines on the management of dyslipidemia showed that the criteria used to initiate lipid-lowering medication in the United States are likely to lead to an earlier initiation of medication compared with Europe [42], which indirectly reduces opportunities to improve diet. This difference could be mitigated in the near future as recommendations have been made to lower treatment thresholds to increase the use of statins for primary prevention of CVDs in European countries [43]. Further research dedicated to between-country differences in CVD risk management is therefore needed to identify systemic facilitators and barriers to the complement approach and to determine how changes in guidelines influence the diet–medication relationship and CVD prevention.

This review needs to be interpreted in the context of strengths and limitations. First, the work we conducted was not limited to a specific design or methodological approach, which allowed us to obtain a broad perspective of the literature on the relationship between medication use and diet in CVD prevention. Including all methodological approaches allowed us to identify strengths and weaknesses associated with the diverse methodologies as well as specific patterns, such as the differences between Europe and the United States, that will fuel further research on this topic. The downside of this strategy, however, has affected our appraisal of the diet–medication relationship, as explained above. Another strength of this work is that multiple health professionals (that is, registered dietitians, pharmacists, registered nurse, medical doctor, and pharmacologist) composed the group of authors, and each provided expertise-specific feedback regarding the directions future research on this topic should focus.

In conclusion, the current literature on the relationship between the lipid-lowering/antihypertensive medication use or initiation and the diet in CVD prevention provides an incomplete perspective on how preventive pharmacological approaches may influence diet. Only 3 studies assessed intraindividual changes in diet associated with medication initiation or use, but none assessed potential changes in diet prior to medication initiation or how the dynamics between medication use and diet influences CVD risk. Still, by evaluating whether lipid-lowering and antihypertensive medications were used as complements to heart-healthy diets, we identified potential between-country differences in the diet–medication relationship. Overall, this work highlights the need for further prospective studies and cross-country comparisons assessing intraindividual changes in diet associated with medication initiation and use, and how these dynamics influence the CVD risk in the long-term. Such work is needed for evidence-based policies systematically favoring the complement approach to optimize medication use in CVD prevention.

## Author disclosures

The authors report no conflicts of interest.

## Author contributions

The authors’ responsibilities were as follows–JPDC: designed the research; CD, MCD: conducted the research; CD, MCD, JPDC: analyzed the data; CD, JPDC: wrote the paper; JPDC: had primary responsibility for the final content; OB, AB, AG, LG, J Leclerc, J Lefebvre, AZ: reviewed the manuscript and provided feedback; and all authors: read and approved the final manuscript.

## Conflict of interest

The authors report no conflicts of interest.

## Funding

No funding was received for this study. CD is the recipient of master’s scholarships from the Fonds de Recherche du Québec – Santé and the Canadian Institutes of Health Research. MCD is the recipient of a Mitacs Acceleration Master’s scholarship. AB is the recipient of a master’s scholarship from Diabète Québec. AG, JL, and JPDC are research scholars of the Fonds de Recherche du Québec–Santé.

## Data availability

All data supporting the current work are available in the tables.

## Appendix A Supplementary data

Multimedia component1 Multimedia component1
