# Supplementary material for: Are Lipid-Lowering and Antihypertensive Medications Used as Complements to Heart-Healthy Diets? A Scoping Review
Source: Adv Nutr. 2023 Apr 29;14(4):870–84. doi: 10.1016/j.advnut.2023.04.010 (PMC10334141; doi:10.1016/j.advnut.2023.04.010)
Supplement: Multimedia component1 [file mmc1.docx]

**Online supplementary material**

**Are lipid-lowering and antihypertensive medications used as complements to heart-healthy diets? A scoping review.**

Clémence Desjardins, Marie Cyrenne-Dussault, Olivier Barbier, Amélie Bélanger, Anne Gangloff, Line Guénette, Jacinthe Leclerc, Jean Lefebvre, Arsène Zongo, Jean-Philippe Drouin-Chartier

**Supplemental Table 1: Search strategy**

| Database | Steps | Search terms | Results (n) |
| --- | --- | --- | --- |
| MEDLINE (via PubMed) | 1. LL medication | (statin) OR (statin users) OR (statin initiation) OR (lipid-lowering drugs) OR (lipid-lowering medication) OR (lipid-lowering drugs users) OR (lipid-lowering drugs initiation) OR (lipid-lowering medication users) OR (lipid-lowering medication initiation) AND (humans[Filter]) | 56,692 |
|  | 1. AH medication | (antihypertensive) OR (antihypertensive drugs) OR (antihypertensive medication) OR (antihypertensive drugs initiation) OR (antihypertensive drugs users) OR (antihypertensive medication initiation) OR (antihypertensive medication users) OR (beta-blockers) OR (ACE inhibitors) OR (calcium antagonists) OR (diuretics) OR (ARB) AND (humans[Filter]) | 320,962 |
|  | 1. Diet | (lifestyle) OR (lifestyle changes) OR (lifestyle modification) OR (lifestyle habits) OR (diet) OR (diet changes) OR (diet modification) OR (diet habits) OR (eating habits) OR (eating pattern) AND (humans[Filter]) | 589,713 |
|  | 1. Combination | #1 AND #2 AND #3 | 934 |
| Embase | 1. LL medication | statin OR (statin AND users) OR (statin AND initiation) OR ('lipid lowering' AND drugs) OR ('lipid lowering' AND medication) OR ('lipid lowering' AND drugs AND users) OR ('lipid lowering' AND drugs AND initiation) OR ('lipid lowering' AND medication AND users) OR ('lipid lowering' AND medication AND initiation) | 60,681 |
|  | 1. AH medication | antihypertensive OR (antihypertensive AND drugs) OR (antihypertensive AND medication) OR (antihypertensive AND drugs AND initiation) OR (antihypertensive AND drugs AND users) OR (antihypertensive AND medication AND initiation) OR (antihypertensive AND medication AND users) OR 'beta blockers' OR (ace AND inhibitors) OR (calcium AND antagonists) OR diuretics OR arb | 264,189 |
|  | 1. Diet | lifestyle OR (lifestyle AND changes) OR (lifestyle AND modification) OR (lifestyle AND habits) OR diet OR (diet AND changes) OR (diet AND modification) OR (diet AND habits) OR (eating AND habits) OR (eating AND pattern) | 1,083,772 |
|  | 1. Combination | #1 AND #2 AND #3 | 1,088 |
| Web of Science | 1. LL medication | TOPIC: (statin) *OR* TOPIC: (statin users) *OR* TOPIC: (statin initiation) *OR* TOPIC: (lipid-lowering drugs) *OR* TOPIC: (lipid-lowering medication) *OR* TOPIC: (lipid-lowering drugs users) *OR* TOPIC: (lipid-lowering drugs initiation) *OR* TOPIC: (lipid-lowering medication users) *OR* TOPIC: (lipid-lowering medication initiation) | 43,452 |
|  | 1. AH medication | TOPIC: (antihypertensive) *OR* TOPIC: (antihypertensive drugs) *OR* TOPIC: (antihypertensive medication) *OR* TOPIC: (antihypertensive drugs initiation) *OR* TOPIC: (antihypertensive drugs users) *OR* TOPIC: (antihypertensive medication initiation) *OR* TOPIC: (antihypertensive medication users) *OR* TOPIC: (beta-blockers) *OR* TOPIC: (ACE inhibitors) *OR* TOPIC: (calcium antagonists) *OR* TOPIC: (diuretics) *OR* TOPIC: (ARB) | 172,890 |
|  | 1. Diet | TOPIC: (lifestyle) *OR* TOPIC: (lifestyle changes) *OR* TOPIC: (lifestyle modification) *OR* TOPIC: (lifestyle habits) *OR* TOPIC: (diet) *OR* TOPIC: (diet changes) *OR* TOPIC: (diet modification) *OR* TOPIC: (diet habits) *OR* TOPIC: (eating habits) *OR* TOPIC: (eating pattern) | 757,607 |
|  | 1. Combination | #1 AND #2 AND #3 | 345 |
| CINAHL via Ebscohost | 1. LL medication | statin OR statin users OR statin initiation OR lipid-lowering drugs OR lipid-lowering medication OR lipid-lowering drugs users OR lipid-lowering drugs initiation OR lipid-lowering medication users OR lipid-lowering medication initiation | 8,096 |
|  | 1. AH medication | antihypertensive OR antihypertensive drugs OR antihypertensive medication OR antihypertensive drugs initiation OR antihypertensive drugs users OR antihypertensive medication initiation OR antihypertensive medication users OR beta-blockers OR ACE inhibitors OR calcium antagonists OR diuretics OR ARB | 17,298 |
|  | 1. Diet | lifestyle OR lifestyle changes OR lifestyle modification OR lifestyle habits OR diet OR diet changes OR diet modification OR diet habits OR eating habits OR eating pattern | 95,448 |
|  | 1. Combination | #1 AND #2 AND #3 | 99 |

The final search was conducted January 14^th^, 2023
